# Longitudinal Study of the Dynamics of Vaginal Microflora during Two Consecutive Menstrual Cycles

**DOI:** 10.1371/journal.pone.0028180

**Published:** 2011-11-30

**Authors:** Guido Lopes dos Santos Santiago, Piet Cools, Hans Verstraelen, Marijke Trog, Griet Missine, Nabil El Aila, Rita Verhelst, Inge Tency, Geert Claeys, Marleen Temmerman, Mario Vaneechoutte

**Affiliations:** 1 Laboratory for Bacteriology Research, Department of Clinical Chemistry, Microbiology, and Immunology, Faculty of Medicine and Health Sciences, Ghent University, Ghent, Belgium; 2 Department of Obstetrics and Gynaecology, Faculty of Medicine and Health Sciences, Ghent University, Ghent, Belgium; 3 Laboratory Clinical Biology, Ghent University Hospital, Ghent, Belgium; University of Hyderabad, India

## Abstract

**Background:**

Although the vaginal microflora (VMF) has been well studied, information on the fluctuation of the different bacterial species throughout the menstrual cycle and the information on events preceding the presence of disturbed VMF is still very limited. Documenting the dynamics of the VMF during the menstrual cycle might provide better insights. In this study, we assessed the presence of different *Lactobacillus* species in relation to the BV associated species during the menstrual cycle, assessed the influence of the menstrual cycle on the different categories of vaginal microflora and assessed possible causes, such as menstruation and sexual intercourse, of VMF disturbance. To our knowledge, this is the first longitudinal study in which swabs and Gram stains were available for each day of two consecutive menstrual cycles, whereby 8 grades of VMF were distinguished by Gram stain analysis, and whereby the swabs were cultured every 7^th^ day and identification of the bacterial isolates was carried out with a molecular technique.

**Methods:**

Self-collected vaginal swabs were obtained daily from 17 non pregnant, menarchal volunteers, and used for daily Gram staining and weekly culture. Bacterial isolates were identified with tDNA-PCR and 16 S rRNA gene sequencing.

**Results:**

Nine women presented with predominantly normal VMF and the 8 others had predominantly disturbed VMF. The overall VMF of each volunteer was characteristic and rather stable. Menses and antimicrobials were the major disturbing factors of the VMF. Disturbances were always accompanied by a rise in Gram positive cocci, which also appeared to be a significant group within the VMF in general.

**Conclusions:**

We observed a huge interindividual variability of predominantly stable VMF types. The importance of Gram positive cocci in VMF is underestimated. *L. crispatus* was the species that was most negatively affected by the menses, whereas the presence of the other lactobacilli was less variable.

## Introduction

The vaginal microflora (VMF) associated with bacterial vaginosis (BV), the most common cause of vaginal complaints in women of childbearing age [1], [2], has been well studied [3]–[10], but the etiology of BV remains largely unknown [2].

To obtain better understanding of the etiology of BV, better understanding of the dynamics of the VMF in general may be imperative, and this might be best achieved by means of longitudinal studies, combining Gram stain grading of the VMF, wet smear, vaginal pH measurement and culture dependent and independent microbiological techniques, documenting in more detail the dynamics of the VMF during the menstrual cycle (MC).

The vaginal econiche is highly dynamic, subject to cyclical changes during the menstrual cycle, whereby the normal domination of the lactobacilli is frequently challenged [11].

The cyclic fluctuation of oestrogen and progesterone levels during the menstrual cycle influence the environment of the lactobacilli, by affecting the vaginal epithelial cell surface receptor expression, the amount and viscosity of cervical mucus, the amount of vaginal transudate, the glycogen level (which is the major substrate for lactate production by the lactobacilli), vaginal oxygen and carbon dioxide tension, the reduction-oxidation potential, the pH [12], [13], and the vaginal innate immune response [14]. Several studies indicated that *Lactobacillus* (*i.e. L. crispatus, L. jensenii)* growth increases throughout the menstrual cycle, but decreases during the menses when the concentration of non-*Lactobacillus* species (*e.g. G. vaginalis*) is increased [15]–[17]. Besides the endogenous factors, the VMF may also be affected by exogenous factors, such as sexual intercourse [3], [18]–[22], personal hygiene (showering, bathing and intimate hygiene) [23], cigarette smoking [24] and stress [25].

In recent years, the molecular techniques for studying the VMF have advanced seriously [8], [26], [10], [17], [27]–[29], providing more information about this complex microflora. Longitudinal studies, regarding the composition of the VMF, have been carried out previously (File S1) and have relied on Gram stain [30], [15], [31], [21], [16], [32], [22] or molecular techniques [17]. Only two studies assessed the dynamics of the VMF during the MC through culture [30], [16].

Here, we report the results of a longitudinal study, monitoring the VMF of 17 menarchal, nonpregnant Caucasian women over a period of two MCs (during an average of 58 days). Self-collected vaginal swabs were obtained daily by the volunteers, and used for daily Gram staining and weekly culture, whereby the obtained isolates were identified by a molecular method (tDNA-PCR). To our knowledge, this is the first longitudinal study in which swabs and Gram stains were available for each day of two consecutive MCs, and whereby the swabs were cultured every 7^th^ day and identification was carried out with a molecular technique.

Briefly, the general objectives were to assess the presence and quantity of different *Lactobacillus* species during the menstrual cycle, also in relation to the BV associated species, to assess the influence of the menstrual cycle on the different categories of vaginal microflora and to assess possible causes, such as menstruation and sexual intercourse, of VMF disturbance.

## Methods

### Subjects

Women were recruited by the DRUG unit, a phase I clinical trial unit, experienced in recruiting healthy volunteers, by an e-mail based invitation to all healthy women enlisted in the DRUG database and by additional recruitment efforts, *i.e.* mainly adds that were spread in the Ghent University Hospital and university restaurants.

Twenty five, Caucasian (Belgian) women, aged between 18 and 35 years (average 25.7 SD 5.0), could be recruited for this study, after oral and written informed consent. Screening was assessed approximately 14 days prior to the start of the study. Inclusion criteria were: a regular MC and no use of contraception (*i.e.* no use of spermicides or of an intra-uterine device), except condoms. The exclusion criteria were: pregnancy, complaints about (malodorous) vaginal discharge, chronic use of medication, the use of antibiotics, antimycotics and antiprotozoals during the previous two months, a history of vaginal surgery or hysterectomy, pelvic inflammatory disease, recurrent vaginal infections, recent history of vulval irritation, an active vulval or vaginal dermatological aberration, intimate hygiene within the last week before the study and symptomatic candidiasis or a positive *Chlamydia trachomatis* PCR result. After screening, 22 women were included, of which 17 completed the study.

### Study design

This study was approved by the research ethics committee of the Ghent University Hospital, Belgium (EC UZG 2008/439).

At screening, a gynecological examination was carried out, a questionnaire was filled out, a vaginal swab was taken and the vaginal pH was measured with a pH test strip (Macherey-Nagel, Düren, Germany) by the gynecologist (HV). The study nurse (MT) explained the technique for vaginal self-swab specimen collection and provided the volunteers with a detailed information leaflet, depicting the method of self-swabbing. The volunteers were also given the opportunity to test the self-swab method in the presence of the study nurse (MT). The women were instructed to insert a Nylon Flocked Swab (Eswab, Copan, Brescia, Italy) into the vagina to an approximate depth of 8 cm, and to press the swab against the vaginal wall while rotating it a few times. Subsequently they had to withdraw the swab carefully to prevent vulval contamination and place it in the Eswab container, which contained a modified liquid Amies transport medium. At the closing clinical examination, a basic gynaecological exam including colposcopy was performed and the vaginal pH was determined once again.

The daily taken swabs were stored at 4 °C and brought to the Ghent University Hospital at each 7^th^ day. All vaginal swabs (989 in total, ranging from 49 for volunteer #16 to 71 for volunteer #12, with an average of 58 swabs per volunteer) were Gram stained, and every week the most recently collected swab (with highest bacterial viability), was cultured. In total, 178 vaginal swabs were cultured, at 9 moments for a single volunteer, 10 moments for 9 volunteers, 11 moments for 5 volunteers and 12 moments for 2 volunteers, during two MCs.

Each volunteer was asked to keep a diary. Data were collected for: time of swabbing, menses, hygienic habits, *e.g.* tampon use, bathing, showering and intimate hygiene practices, swimming, sexual intercourse (SI) (with or without condom), stress on a Visual Analog Scale (VAS) from 0 to 10 (0 absence of stress and 10 being stressed out), smoking and alcohol intake, use of antibiotics and any complaints.

### Culture

The vaginal swabs were vortexed for 10 seconds, and 75 µl of swab medium was inoculated onto Schaedler agar (Becton Dickinson, Erembodegem, Belgium), Columbia agar (Becton Dickinson), and TMB^plus^ agar (prepared in-house, as described previously [33]. All the agar plates were incubated at 37 °C in an anaerobic chamber (BugBox, LedTechno, Heusden-Zolder, Belgium) for 48 to 72 h.

### Isolation culture, DNA-extraction from isolates and identification of isolates by tRNA intergenic length polymorphism analysis (tDNA-PCR)

From each plate, one colony was picked for every distinct colony type. A total of 2040 colonies were isolated. DNA was obtained by alkaline extraction [34]. Isolates were identified with tRNA intergenic length polymorphism analysis (tDNA-PCR), as described previously [8], [9], [34]–[37]. Isolates for which no clear-cut identification was obtained by tDNA-PCR, were identified by 16 S rRNA gene sequencing.

### Grading of Gram-stained vaginal smears

A volume of 50 µl of the swab medium was used for Gram staining. A total of 989 Gram stained vaginal smears (average of 58 per woman) were scored by four independent assessors (GC, RV, GM and GL), according to the criteria previously described [9]. Briefly, Gram stained vaginal smears were categorized as grade I (normal) when only *Lactobacillus* cell types were present, as grade II when both *Lactobacillus* and BV-associated cell types were present, as grade III when BV-associated cell types were abundant without lactobacilli present, as grade IV when only gram-positive cocci were observed, as grade I-like when gram-positive rods, either quite small and short or otherwise irregularly shaped with curved edges, were predominant and as grade 0 when no bacterial cells were present.

Grade I specimens were further subdivided into grade Ia specimens when only *Lactobacillus crispatus* cell types (plump, mostly short rods) were present, grade Ib when only other *Lactobacillus* cell types were present (smaller or more elongated and less stained than in grade Ia smears) and grade Iab when both *L. crispatus* and other lactobacilli were present.

## Results

### Relation between VMF and clinical symptoms

**Figure 1:**
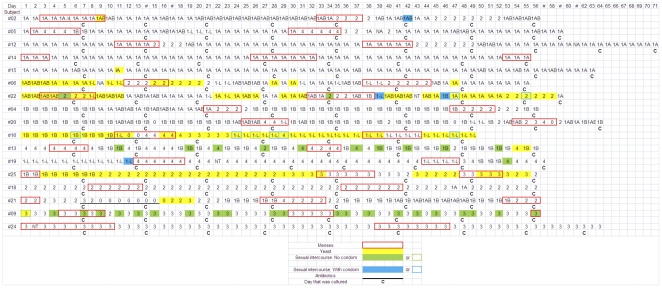
Overview of VMF grades, yeasts, menstrual periods, sexual intercourse and antibiotic usage for the 17 participants. Legend: 1A: grade Ia; 1B: grade Ib; 1AB: grade Iab; 1-L: grade I-like; 2: grade II; 3: grade III; 4: grade IV.

The objective of this study was to document the VMF during two consecutive MCs, by Gram staining of the daily self-sampled vaginal smears and by culturing on a weekly basis. The 17 volunteers could be allocated into two groups, whereby group N(ormal) (n = 9) comprised the women with a predominantly stable normal VMF (grade I) and group D(isturbed) (n = 8) comprised the women with predominantly disturbed VMF (grades I-like, II, III and IV, *i.e.* non-grade I) (Table 1 and Figure 1).

**Table 1:** Chronology of the VMF grades for each subject during two menstrual cycles (see also Figure 1 for details).

	Subject	Age (years)	Screening	Start	M1	Midcycle	M2	Midcycle	M3	Midcycle	End
**Group N Normal VMF**	2	28	Ia	Ia	Ia + GPC→ Iab	Ia → Iab	Iab → II	Iab ↔ II	NA	NA	Iab
	5	24	Ia	Ia	Ia→ IV → Ib + GPC	Ia→Iab→I-like→Ia	Ia→IV	III→II→Ia↔Iab	NA	NA	Iab
	12	26	Ia	Ia	Ia + GPC→ II	II →Ia	Ia + GPC	II →Iab → Ia	NA	NA	Ia
	14	32	Ia	M1	Ia + GPC	Ia	Ia + GPC	Ia	Ia + GPC	NA	Ia
	15	23	Ia	Ia	Ia (No menses)						Ia
	6	25	Ia	Iab + Y	II	II+Y→I-like↔Iab↔Ia +Y	I-like →II	Ia + Y	NA	NA	Ia
	22	24	Ia	Iab + Y	Iab+Y →II+Y→I-like + Y	Iab+Y→Ia↔Iab →Ia+Y↔Iab+Y	Iab+GPC↔II	Iab+Y↔Ia+Y→II+Y	NA	NA	Ia
	4	20	I-like	Ib	Ia+GPC→II	Ib	Ib→II	II→Ib	NA	NA	Iab
	20	34	Ib	Ib	Iab → IV →Iab +GPC	Ib →I-like →Iab	Iab+GPC →II →III →IV →0	II →Iab →Ib	NA	NA	Ib
**Group D Disturbed VMF**	16	26	Ib +Y	Ib +Y	I-like + Y →0 →IV	IV+Y →III+Y →I-like+Y	I-like+Y →I-like	I-like+Y	NA	NA	I-like +Y
	13	22	Ib	IV	IV	Ib ↔ IV ↔ II	IV → II → IV	IV↔ Ib↔ II	NA	NA	Ib
	19	22	Ia	I-like	IV	IV	I-like	III→IV→Ib→IV	NA	NA	IV
	25	18	II	M1	Ib	Ib+Y→II+Y→III+Y	III	III→II+Y	III→III+Y	III+Y→II+Y	II+Y
	18	29	II	II	II	II	II	II→Ia→II	NA	NA	Ia
	21	32	II	M1	II	III ↔II →0→0+Y→II+Y→III+Y	Ib+GPC→II	II→Ib↔ Iab	Ia→II	NA	II
	9	33	II	III+Y	III	III	III	III	III	NA	III
	24	19	III	M1	III	III	III	III	NA	NA	III

Legend: GPC: Gram positive cocci, Y: Yeast; NA: Not applicable, →: changing from grade indicated on the left to grade indicated on the right; ↔: changing back and from between both grades.

Overall, complaints were minor, although half of the women presented with predominantly non-grade I VMF. Only 2 out of 9 women from group N (#20, #22) had complaints, which were minor for subject #20, lasting only 3 days. Subject #22, with pruritus during 10 days, was - together with subject #6 - one of the two women in this group with prolonged presence of yeasts. Five out of 8 subjects from group D had complaints. Subject #9, with grade III VMF during the whole study and with yeast present only the first day, had complaints about pruritus, irritation and redness only during the first three days of the study. Subject #13 had an episode of vaginal bleeding after SI without condom, whereby a grade IV VMF developed after 1 day. She complained about pruritus and irritation on two consecutive days following an event of SI without condom. Subject #18 reported minor complaints of white vaginal discharge on 5 days (1 day grade Ia VMF and 4 days grade II VMF). Subject #19 had complaints for 32 of the 57 days of her study period. Pain in the hymen (at the introitus) accounted for 13 days of which 7 days with intimate hygiene practices (5 days grade I-like and 8 days grade IV), 9 days of unspecified vaginal pain of which 3 days with intimate hygiene practices (grade IV), 8 days of pain accompanied by vaginal discharge of which 5 days with intimate hygiene practices (grade IV), and 2 days of irritation of which 1 day with intimate hygiene practices. Subject #21 with yeast on four days, had complaints about pruritus on two of these days. Both the subjects with yeast during the entire study period (#16 and #25), reported no complaints. In brief, we could not establish a relationship between the presence of yeasts and complaints, but complaints were predominantly accompanied by grade II or IV VMF, but not grade III. For example, two women (#9 and #24) had grade III, i.e. BV-associated VMF throughout the entire study period, but showed no (#24) or only minor (#9) complaints and were not clinically diagnosed as having BV.

### Evaluation of the VMF status based on vaginal pH

The vaginal pH was determined at screening and at conclusion of the study. The average pH was 3.7 (SD 0.2) in grade Ia (n = 13), 4.0 (SD 0.54) in grade Ib (8), 3.9 (SD 0.35) in grade Iab (2), 4.2 (SD 0.71) in grade I-like (3), 4.6 (SD 0.34) in grade II (5) and 5.2 (SD 0.17) in grade III (3).

### Evaluation of VMF status based on Gram stained vaginal smears

All of the women in group N entered the study with a normal VMF and maintained this largely for the entire study period, although 2 subjects (#6 and #22) were colonized during prolonged periods with yeasts. Major disturbances of the VMF in this group occurred during the menses.

In group D, only 79 out a total of 449 analysed Gram stain smears, were smears representative of normal VMF, i.e. 2 grade Ia, 71 grade Ib and 6 grade Iab (only found between the grade Ib instances). Group D consisted of 8 women with various types of disturbed VMF. Two women in this group (#9 and #24) had exclusively grade III VMF, with high numbers of Gram positive cocci (GPC), two women (#18 and #25) had almost exclusively grade II VMF. The stability of the VMF of #16 and #21 was variable, although with a predominance of I-like VMF for #16 and of grade Ib and II VMF for #21, but this variability was possibly a result of the usage of antimicrobial agents. Two women (#13 and #19) had predominantly grade IV VMF, switching with Ib VMF on short notice for #13 or with I-like VMF for prolonged periods for #19. Two women (#16 and #25) in this group were colonized by yeast for almost the full length of the study.

Grade Ib was present in 5 out of 8 women in group D and 4 out of 9 women in group N of which 2 women (#4 and #20) had grade Ib throughout most of the study period. Interestingly, all transient grade Ib episodes were preceded or followed by grade II or IV VMF, while no shifts between grade Ib and grade III were observed.

In conclusion, based on Gram staining (Table 1 and Figure 1), it can be concluded that most women had predominantly a stable type of VMF during the whole study period, except during and/or briefly after a disturbing event, such as the menses (see below).

### Presence of different species based on culture

Using culture, performed once per week, on average 10 times per subject, the presence of some predominant vaginal species could be established. *L. crispatus* was found in 7 of the 9 women of group N, during all phases of the MC, including the menses, and in all VMF grades, except grade III VMF, whereas in group D, *L. crispatus* colonized only 2 out of 8 women during all phases of the MC, and was completely absent during the menses. Also *L. jensenii* was found in 6 of the 9 women in group N and during all phases of the MC. *L. jensenii* was the only *Lactobacillus* species cultured from grade III VMF samples in this group. In group D, *L. jensenii* was present in volunteer #25 during all phases of the MC and occasionally in volunteer #18. *L. gasseri* was found in 2 women (#4 and #22) from group N during all phases of the MC and in subjects #13 (second MC only) and #18 (at 2 moments only) of group D. *L. iners*, although also found in subjects of group N (5/10 times for #4, 7/11 for #15, 3/11 for #20 and 1/11 for #22), was more predominantly present in 6 women of group D, in 4 of them during all phases of the 2 MCs.

*L. vaginalis* was cultured for 3 women (#4, #5 and #22) from group N and 4 women from group D (#9, #16, #18 and #25), but never during the menses. *L. coleohominis* colonized rather permanently (10 out of 11 culture moments) one volunteer (#15), *i.e.* the subject without menses during the study period, with a constant grade I VMF for the entire study period and also with *L. crispatus* and *L. iners* at 7 out of 11 culture moments.

**Figure 2:**
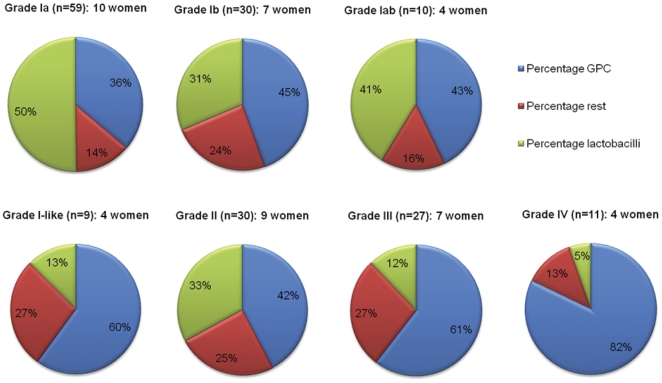
Percentages of vaginal swabs, culture positive for lactobacilli, gram positive cocci (GPC) and other species for different VMF grades.

*G. vaginalis* was present during all stages of the MC, including the menses, in 3 of the 9 women from group N (#6, #12 and #22) (9 of the 98 culture moments), but more frequently in group D (6 of the 8 women, 37 of the 80 culture moments). *A. vaginae* was cultured only 3 times from 2 women of group D (#9 and #21). GPC were cultured in both groups, predominantly during the menses and after SI, with: *Peptostreptococcus asaccharolyticus* (43.3%) and *Streptococcus anginosus* group (40.4%) being the most prevalent GPC. Based on culture results, the GPC appear to be an important microbial component of the normal VMF, but even more of disturbed VMF (Figure 2, File S2).

In general, there is a downward trend in the presence of lactobacilli and an upward trend in the presence of GPC from grade Ia over grade Iab to grade Ib, respectively. Interestingly, the distribution percentages of the 3 major species groups are almost equal for grade Ib and grade II VMF, and for grade III and I-like VMF (Figure 2).

### Comparison of culture results per VMF grade

The bacteria listed in File S2 were present in at least 10% of the samples of at least one of the VMF grades. Among the grade Ia VMF samples, *L. crispatus* was the major constituent present in 76.3% of the samples, followed by *L. jensenii* and the GPC, *i.e. S. anginosus* group and *P. asaccharolyticus*. *L. iners* and *L. gasseri* were not commonly found in grade Ia samples.

In grade Iab, *L. crispatus* was also the major constituent, followed by *L. jensenii*. The GPC *P. asaccharolyticus, S. epidermidis* and *S. anginosus* group were observed as well in an important number of samples. *L. iners* was more prominent in grade Iab samples compared to grade Ia samples.

In grade Ib VMF, *L. iners* and *P. asaccharolyticus* were the major constituents, followed by *L. gasseri* and *Alloscardovia omnicolens.*

Grade I-like samples were characteristically dominated by GPC, *i.e. E. faecalis*, *P. asaccharolyticus* and *S. anginosus* group. It was the only grade where bifidobacteria, *i.e. Bifidobacterium dentium* and *B. breve,* were isolated in more than 10% of the samples.

For grade II VMF, *G. vaginalis* and *P. asaccharolyticus* were both cultured from 60% of the samples. All lactobacilli listed in File S2 were present in grade II samples with *L. jensenii* being the most prevalent, followed by *L. crispatus*. Grade II VMF was mostly a mixture of *G. vaginalis*, GPC and lactobacilli.

In grade III samples, *S. anginosus* group, *G. vaginalis* and *P. anaerobius* were the most frequently cultured bacteria. The overall presence of GPC was high as well. The only lactobacilli present above the 10% threshold were *L. iners* and *L. jensenii*.

Grade IV VMF was dominated by *A. omnicolens* and GPC, more specifically, *S. anginosus* group, *E. faecalis* and *P. asaccharolyticus*. *G. vaginalis* and lactobacilli (*L. crispatus, L. gasseri* and *L. iners*) were present in lower numbers, just below the 10% threshold.

Table 2 provides an overview of the most frequently cultured species found in the different VMF grades from women of both groups N and D. Only species found in at least 4 of the cultured samples were included.

**Table 2:** Species found in at least 4 cultured samples during the course of 2 MCs, with indication of their presence in the VMF of groups N and D.

Species cultured	Ia	Iab	Ib	I-like	IV	II	III	0	N (9)/D (8)	Present during menses (N/D)
*Aerococcus christensenii*							D		0/1	0/Y
*Actinomyces neuii subsp. anitratus*	N								1/0	No menses
*Alloscardovia omnicolens*	D		N/D		D		D		1/2	Y/Y
*Atopobium parvulum*							D	D	0/1	0/Y
*Anaerococcus tetradius*							D		0/2	0/Y
*Bifidobacterium dentium*				D					0/1	0/N
*Escherichia coli*			N	N	N		N		1/0	Y/0
*Enterococcus faecalis*	D		D	D	D		D	D	0/2	0/Y
*Finegoldia magna*			D	D		D	D	D	0/2	0/Y
*Gardnerella vaginalis*	N	N	D		D	D	D		1/6	Y/Y
*Klebsiella pneumoniae*	D			D	D		D		0/1	0/N
*Lactobacillus coleohominis*	N								1/0	No menses
*L. crispatus*	N	N	N/D	N	N	N/D	D		7/2	Y/N
*L. gasseri*	N/D	N	N/D		D	N/D			2/2	Y/Y
*L. iners*	N	N	N/D		D	D	D		2/5	N/Y
*L. jensenii*	N	N	N	N		N/D	N/D		6/1	Y/Y
*L. vaginalis*		N	N/D			D	D		2/2	N/N
*Peptostreptococcus anaerobius*	N		D			N/D	D		1/3	Y/Y
*P. asaccharolyticus*	N/D	N	N/D	N	N/D	N/D	D		6/5	Y/Y
*Propionibacterium avidum*	N	N							1/0	N/0
*Prevotella amnii*				D			D		0/1	0/Y
*P. bivia*				D			D		0/2	0/Y
*P. disiens*			N						1/0	Y/0
*Streptococcus agalactiae*	N	N				N			1/0	Y/0
*S. anginosus* group	N	N	D	D	N/D	N/D	D		3/6	Y/Y
*Staphylococcus aureus*			D			D	D		0/1	0/Y
*S. epidermidis*	N	N	N/D			N/D			3/1	Y/Y
*Veillonella parvula*				D					0/1	0/N

Legend:

D: Present only in group D (women with non-grade I VMF)

N: Present only in group N (women with grade I VMF)

N/D: Present in both groups N & D

Y: yes; N: no; 0: no women of this group with this species

### Influence of disturbing factors on VMF

#### 1. Menses

There were 36 documented menstrual periods (MPs) for 16 women in this study (subject #15 had no menses during the study), of which 29 were cultured for 15 women, 8 from group N (15 cultured MPs) and 7 from group D (14 cultured MPs). Seven of the 36 MPs (including both MPs documented for subject #18, group D), could not be cultured, because these MPs did not fall at the end of each weekly sampling period, *i.e.* on the day that was cultured. In total, 3 MPs were cultured for one subject (#9), 2 MPs were cultured for 12 women and one for two women (#5 and #19).

As mentioned above, most subjects had a rather stable VMF during the study period, *i.e.* each woman presented with a predominant type of VMF during the whole study period (Table 1 and Figure 1). Nevertheless, in group N, the VMF changed during 12 out of 17 of the documented MPs to become more dominated by GPC, *i.e.* changing to grade II (n = 8) or IV VMF (n = 4), and even for the 5 MPs (subjects #2 (1 MP), #12 (1 MP) and #14 (3 MPs) for which normal VMF remained predominant during the menses, a rise in GPC on Gram stain and by culture was noticed. Subject #12 developed grade II VMF only at the last day of MP1 and immediately after MP2, lasting 4 respectively 5 days.

In group D, 15 of the 20 MPs were characterized by a rise in GPC (Table 1), but the non-grade I VMF, already present before the menses, remained unchanged throughout and after the menses.

The disturbance of the VMF associated with menstruation in group N (12 events, 8 subjects) restored mostly fast, *i.e.* 1.8 days on average (range 0–7 days).

The 4 women, 2 from group N (#6 and #22) and 2 from group D (#16 and #25), with a total of 9 documented MPs, for whom yeast could be observed as part of the VMF during the study, were predominantly free of vaginal yeasts during the menses, whereafter they were recolonized with yeast.

**Figure 3:**
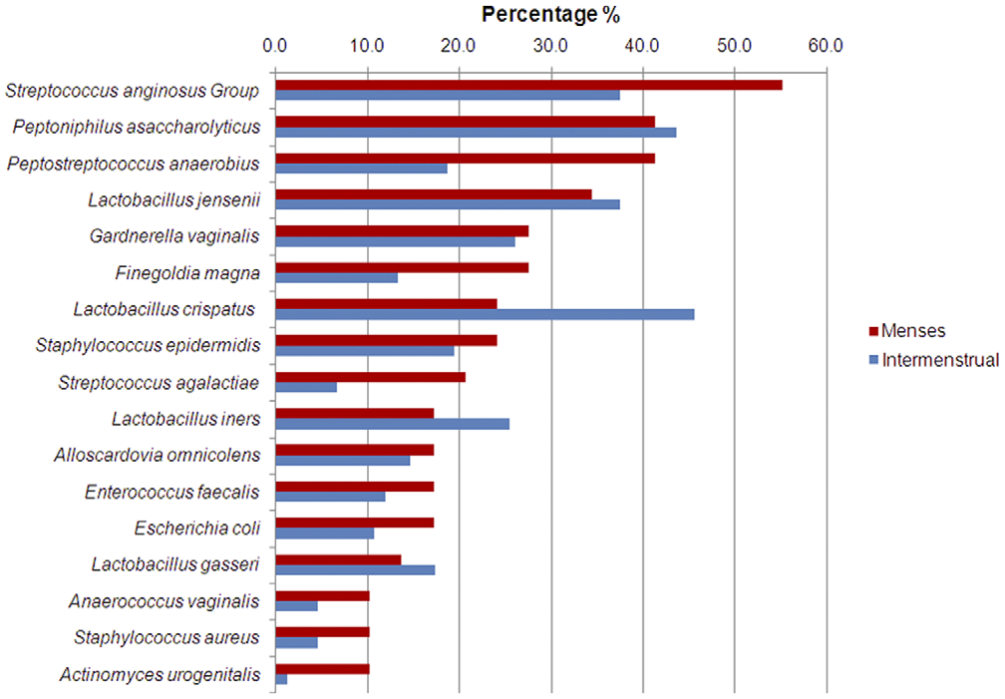
Percentages of vaginal swabs, culture positive for species present in at least 10% of the 29 samples collected during menstruation. Legend: Red: percentage of 29 menstrual samples culture positive for this species.Blue: percentage of 149 intermenstrual samples culture positive for this species.

Figure 3 presents the prevalence of the bacterial species, present in at least 10% of the total number of culture moments during the 29 MPs, irrespective of the VMF grade. The most prevalent species during the menses in comparison to outside the menses were the *S. anginosus* group, *P. anaerobius, Finegoldia magna* and *Streptococcus agalactiae.* The presence of *L. crispatus* was strikingly lower during the menses, whereas there was little difference during and outside the menses for the other lactobacilli and for *G. vaginalis.*

In general, the number of women with samples positive for lactobacilli dropped during the menses (Figure 3). *L. iners* and *L. vaginalis* were not cultured during the menses in group N, whereas *L. crispatus* and *L. vaginalis* were absent during the menses in group D.

#### 2. Sexual intercourse (SI)

The VMF was documented around a total of 47 SI events, 7 in group N (subjects #2 and #22) and 40 in group D (subjects #9, #13, #16 and #19). The possible influence on the VMF could not be determined for 38 SI events (33 from group D and 5 from group N), because the VMF was already non-grade I, because SI occurred during the menses or was followed by another day with SI.

In group N, the only two episodes of SI with condom that were assessable (for #2 and #22) did not seem to influence the VMF.

In group D, there were 6 SI events with condom (for #16 and #19), but due to already disturbed VMF, only one was assessable and did not influence the grade Ib VMF. A total of 6 SI events without condom were assessable, all for subject #13. The grade Ib VMF did not change (n = 1) or changed to grade IV VMF (n = 4) or to grade II VMF (n = 1), after 1 to 2 days and the disturbance lasted for 1 to 2 days. However, it cannot be concluded whether this was due to SI, because the VMF of this subject switched between Ib, II and IV continuously during the follow up period.

#### 3. Antibiotic therapy

Two women (group D) used antimicrobial agents (not specified) during the course of this study, *i.e.* during the first menses (#16), and for one week, just after the first menses (#21). On Gram stain, no bacteria were visible during treatment (grade 0), but culture revealed the presence of *F. magna* in both volunteers, with in addition *A. parvulum*, *E. faecalis* and *S. epidermidis* in volunteer #16, and of *E. coli* in volunteer #21.

Subject #16, who started the study with grade Ib, including *L. vaginalis, L. iners* and yeast, followed an antifungal cure that eradicated the yeast and most of the bacterial cells. After the cure, a VMF ranging from grade IV to grade III, and finally grade I-like was established, with the yeast reemerging, but without lactobacilli. The species present during grade 0, mentioned above, persisted after treatment, for the rest of the study period.

Subject #21 started the study with grade II VMF (including *L. crispatus, L. iners* and *L. gasseri*), and the antibiotic treatment eradicated most of the bacterial cells. After the cure, the yeast reappeared and after a day of grade 0 VMF, a grade II VMF (including *L. iners*) reemerged, but after 6 days a grade Ib/Iab VMF (including *L. crispatus, L. iners* and *L. gasseri*) installed for the remainder of the study (with exception for the menses).

#### 4. Influence of behavioural factors/personal hygiene on VMF

Showering or bathing did not disturb the VMF. Volunteer #14, who bathed daily, and volunteer #12 who showered daily, had a stable Ia VMF during the whole study. Five women used wash solutions (not specified) for intimate hygiene. In group N, volunteers #04, #12 and #22 practiced intimate hygiene on a total of 20, 2 and 2 days respectively, but this did not influence the VMF. In group D, volunteers #19 and #24 practiced intimate hygiene on a total of 25 and 5 days respectively, but due to the already disturbed nature of their VMF, possible disturbing influences could not be assessed. Usage of tampons was comparable among women with predominantly normal VMF (6 out of 9) and women with predominantly disturbed VMF (6 out of 8). No effect on the VMF was noted for group N and none could be assessed for group D.

The data for smoking and alcohol intake were too limited for conclusions. The average stress factor in both groups was comparable (3.0 for group N, 3.9 for group D).

## Discussion

### General setup

In this study, we enrolled 17 menarchal, non-pregnant Caucasian subjects, who self- swabbed the vagina daily during two consecutive menstrual cycles (MCs) and whereby Gram stains were carried out each day and culture was performed each week.

For grading the Gram stains, the modified Ison and Hay criteria [9] were used because this scoring system takes into account the clear differences within grade I VMF, on the basis of different *Lactobacillus* cell types, and recognizes the presence of an extra grade, *i.e.* grade I-like, as explained in the Materials & Methods section. A strong correlation was found between grade Ia and the presence of *L. crispatus* and between grade I-like and the presence of bifidobacteria [9].

In summary, the following overall observations could be made.

Although inclusion and exclusion criteria were aimed at enrolling women with normal VMF, seven out of 17 women had complaints of for instance irritation and pruritus during the study. Although vulvovaginal complaints are quite common among fertile women, they are often limited to transient discomfort [38], as was the case in this study.

Also, despite the inclusion criteria, only 9 of the women presented with predominantly normal VMF (*i.e.* grades Ia, Ib and Iab, group N), whereas 8 others had predominantly non grade I VMF (grades I-like, II, III and IV, *i.e.* disturbed VMF, group D). A first conclusion is that there is a huge interindividual variability of predominant VMF present. For example, the continued presence of a *Lactobacillus* species, infrequently encountered in the vagina, *i.e. L. coleohominis*, was observed for one volunteer (#15), the one subject without menses during the entire study period, and with a constant grade I VMF.

Another observation is that, despite the huge interindividual diversity, the VMF of most subjects is rather stable (Figure 1).

Thoma *et al.* (2011), who sampled 255 sexually experienced women on a weekly basis during two years, found that normal VMF persisted for 76.1% of the women, BV persisted in 73.6% of the women, whereas intermediate VMF had similar probabilities of progression (36.6%), resolution (36.0%) and persistence (27.4%) [39].

Priestley *et al.* (1997) found 9 women out of 26 with the same grade of VMF throughout the study, whilst Keane *et al.* (1997) recorded 14 out of 21volunteers with consistently normal or consistently disturbed VMF. Schwebke *et al.* (1999) reported 11 out of 51 women with a stable normal VMF and Brotman *et al.* (2010) found 9 out of 33 women with a normal VMF throughout the study. The larger proportion of women with predominantly stable VMF in our study might be explained by different populations studied or by different exclusion criteria used.

However, the overall stability of VMF is not in disagreement with our previous studies, reporting a large degree of correspondence between rectum and vagina, not only at the species and strain level [34], [40], but also of the bacterial loads [41], indicating that the rectum might serve as the reservoir for restoring the predominant VMF, possibly explaining the recovery capacity of the VMF after disturbing events.

Although we generally found overall stability of the VMF during the menstrual cycle, sudden changes could be established in this study, as was also reported previously [30], [21]. This indicates that occasional sampling may miss episodes of BV or candidiasis (e.g., five women were only sporadically, *i.e.* 1–4 days, positive for yeast) and on the other hand may overemphasize the importance of an occasional single positive swab.

Comparison of culture results between groups N and D in the present study led to the following observations: in group N, *L. crispatus* and *L. jensenii* were the most frequently cultured species, confirming previous studies [9], [42], [27], and they were present during all phases of the MC.

In general, *L. crispatus* and *L. jensenii* were the major constituents of grade Ia and Iab, and *L. iners* and *L. gasseri* were predominant in grade Ib. *L. crispatus* and *L. jensenii* were also frequently cultured from grade II VMF samples, whereas *L. iners* was the predominant *Lactobacillus* species in grade III VMF. These results are largely in accordance with our previous (cross sectional) studies [9], [34], [10]. *L. crispatus* was the species that was most negatively affected by the menses, the presence of the other lactobacilli did not change much.

Besides lactobacilli, also the GPC, especially the *Streptococcus anginosus* group, *Peptostreptococcus anaerobius* and *P. asaccharolyticus*, were cultured from 20–40% of samples from all grades, with predominant presence during menses, after SI and during and after antibiotic treatment.

Bartlett *et al.* (1977) and Johnson *et al.* (1985) already showed that besides lactobacilli, GPC are important members of the VMF [43], [44]. The importance of this group of bacteria in VMF, with the exception of group B streptococci, may have been underestimated.

The lowest GPC numbers were found in grade Ia and the highest numbers in grade Ib, I-like, II, III and IV VMF (Figure 2). Despite a difference in Gram stain appearance, the distribution between GPC, lactobacilli and the remainder of the cultured species was strikingly similar between grades Ib and II and between grades I-like and III (Figure 2). For grades Ib and II, this might be in agreement with the observation that for the women of our study population periods of grade Ib VMF frequently switched with periods with VMF grade II, but no such temporal link could be established between grade I-like and grade III VMF.

The Gram stain results, especially from women of group D, indicated that grade Ib may be an intermediate VMF, because it often followed or preceded grade II or IV VMF. This might explain the increased frequency of bacterial species in grade Ib VMF that are also more prominent in grade II and IV VMF (File S2). However, grade Ib has, in comparison to the other VMF grades (File S2), the highest percentage of *L. iners*, which is also important in grade III VMF and has been suggested to be more resilient to conditions associated with disturbed VMF [28]. Previously, we reported that pregnant women with grade Ib are more likely to evolve to a disturbed VMF towards the end of pregnancy, compared to women with grade Ia or Iab [45].

In conclusion, the increased presence of GPC seems to be the most characteristic feature in common to all disturbances of the VMF and could be explained as an absolute increase of the GPC loads or as the already present GPC becoming more easily detectable, due to the disappearance of the lactobacilli. Possibly, stress conditions can reduce the number of lactobacilli, whereby the GPC already present in the VMF will increase and the GPC from the rectum (colon) will also get the opportunity to colonize the vagina. In the studies by El Aila *et al.* [34], [40], the most abundant species that could be cultured from rectal swabs were the *S. anginosus* group, *F. magna*, *Peptostreptococcus* spp, *Peptoniphilus indolicus* and *E. faecalis*. These species were also found vaginally, but to a lesser extent [34], [40].

In this study, the presence of *G. vaginalis* in both groups of women, during all stages of the MC, including the menses, confirms previous observations that *G. vaginalis* is a natural constituent of the vaginal microflora [10], [46], however more prominently present in grades II and III VMF.

### Influence of disturbing factors on VMF

Our data, based on small numbers of assessable SI events, do not indicate strong influence of SI on the VMF. Also, subject #24 reported no SI events, but her VMF was continuously grade III. However, the difference between the number of SI events (7 in group N vs. 40 in group D), and the number of SI events without condom in both groups (2 in group N vs. 34 in group D) could be in agreement with the findings of Cherpes *et al.* (2008), who demonstrated that the chance of BV acquisition is correlated to the frequency of SI [3]. Although BV related microbes can be transmitted sexually [47], we previously suggested that BV is primarily a sexually ‘enhanced’ condition, *i.e.* disturbance of the VMF is not a consequence of intercourse with multiple partners or with a new partner (indicative for sexual transmission) in the first place, but of increased sexual activity in general [18].

A major impact on the VMF was observed by usage of antimicrobial agents during the study, as was done by two volunteers (#16 and #21). Again, during treatment, GPC were the main components of the VMF. For volunteer #16, the lactobacilli present before the cure remained absent afterwards. Assuming that the antibiotics also affect the rectal microflora, this might suggest that antibiotic treatment on its own is insufficient to restore a woman's VMF and that additional therapeutics, *e.g.* probiotics with vaginal lactobacilli, might be useful for restoring *Lactobacillus*-dominated VMF, as previously indicated by several studies [48]–[51].

In contrast to the observation of Klebanoff *et al.* (2010) [23], showering, bathing or intimate hygiene products did not seem to influence the VMF in this study.

### Conclusions

We assessed the presence of various bacterial species during the menstrual cycle, during the menses and after other, possibly VMF-disturbing events.

It can be concluded from this study that the VMF of women with predominant grade I VMF can shift to more GPC dominated VMF and to other non grade I VMF because of disturbing events such as menses and antibiotic usage. This is in correspondence with the general view developed by Hay (2005), stating that lactobacilli are in a continuous effort to acidify the vaginal econiche after the different disturbing events that may occur in this dynamically changing environment [11]. Whether and how easily they succeed in maintaining predominance, providing colonisation resistance to anaerobes, appears to be strongly different between individual women.

The differences between women might be caused by genotypic differences between their vaginal lactobacilli (different *Lactobacillus* species, different strains of *L. crispatus*, differences in production of lactic acid, hydrogen peroxide and bacteriocins), between women (differences in hormone metabolism (influenced also by contraception) and innate immunity), and between the match of certain strains to certain hosts, *e.g.* influencing adherence capacities of the lactobacilli.

Whether or not lactobacilli succeed to remain predominant will largely depend on a combination of two factors, *i.e.* the ease and speed with which the lactobacilli can acidify/colonize the vaginal econiche after a disturbing event (see above) and the number of disturbing events they are confronted with.

The paradigm that a *Lactobacillus* dominated VMF is necessary to ensure the absence of BV symptoms may also need some revision. The study of Priestley *et al.* (1997) raised doubts about what should be regarded as normal VMF. Apparently, asymptomatic women can be devoid of lactobacilli [52], but still be colonized by lactic acid and/or hydrogen peroxide producing microorganisms, such as *Atopobium, Megasphaera* and/or *Lepotrichia* species, *Streptococcus* species and *Weissella* species [53]–[56].

Ravel *et al.* (2011) suggest that a more refined definition of the different bacterial communities normally found in healthy women is needed and the differences between individuals should be taken into account in risk assessment and disease diagnosis [29]. Indeed, the microscopically observable differences after Gram stain of vaginal swabs, also between grade I samples, do correspond with different cultivable species present and this study confirms that further categorization of grade I samples, as grade Ia, Iab, Ib (and I-like) may be important, *e.g.* because grade Ib might be an intermediate between Ia or Iab and II or IV.

An important observation of this study is the possible existence of an equilibrium between lactobacilli and GPC in normal VMF, which can be disturbed by menses and SI. The role of GPC in the overall dynamics of the VMF deserves further study.

### Limitations of this study

Our primary limitation is the small study population. Longitudinal studies requiring daily efforts of participants are difficult to carry out, because many volunteers will fail to complete the study. Instead of processing limited numbers of samples from many women, we studied many samples from a relatively small number of women. Whilst most longitudinal studies only did Gram staining, we combined it with culture and identification to the species level.

A second limitation may be that the women were recruited from the university hospital and the student population, possibly introducing volunteering bias and other types of selection bias, in as much that the ultimate sample was skewed towards young, sexually active, higher educated women.

Another limitation may be the fact that samples were obtained by the subjects, which holds a risk for vulval contamination. However, studies like this are impossible without self-sampling. Moreover, it has been shown that there is no difference between self-swabbing and swabs taken by the clinician [57], [17], [58].

A correlation between the presence of certain bacterial species and clinical symptoms could not be established, because we aimed at excluding women with symptoms in the first place.

Diaries were not always informative enough, e.g. the brands of intimate hygiene products or the antibiotics used were not noted. Despite the lack of information on the type of antibiotics used, we found it interesting to document the associated VMF changes on a day-to-day base, as was not yet published before to our knowledge.

No statistics could be performed on the limited metadata. The data obtained did not allow for any meaningful statistical analyses like trend analysis or multivariate analysis.

The most important limitation may be the fact that this is a culture-based study. Therefore, fastidious organisms (e.g. *Atopobium vaginae*) or organisms for which the specific culture media are needed (*Mycoplasma hominis* and *Ureaplasma urealyticum*) are underreported or absent. Only one truly longitudinal study, using molecular techniques (qPCR), has been carried out [17]. This enables quantification of the bacteria, independent of culture, but is limited to species for which specific PCRs are included. Ultimately, an ideal study to fully assess the dynamics of the VMF would be a longitudinal study, comprising at least one menstrual cycle, with daily sampling, combined with extensive and detailed metadata on subjects' behaviors, with scoring of Gram stained vaginal smears by the modified Ison and Hay system [9], with culture combined with species level identification of the isolates, as in this study, and with deep sequencing based analysis [29].

## Supporting Information

File S1 **Summary of published longitudinal studies regarding the composition of the VMF.**
(PDF)

File S2 **Bacterial species cultured from at least 10% of all samples of at least one of the VMF grades.**
(PDF)
