# Supplementary material for: Longitudinal Study of the Dynamics of Vaginal Microflora during Two Consecutive Menstrual Cycles
Source: PLoS One. 2011 Nov 30;6(11):e28180. doi: 10.1371/journal.pone.0028180 (PMC3227645; doi:10.1371/journal.pone.0028180)
Supplement: File S1 — Summary of published longitudinal studies regarding the composition of the VMF. (PDF) [file pone.0028180.s001.pdf]

Supporting Information file 1.

Authors	Inclusion criteria	Sample	Subjects	Techniques	Grading	Period	Sampling frequency	Principal findings
**Priestley *et al.* (1997) [1]**	No specific inclusion criteria are given	Vaginal self swabs	26	Gram stain<br>Vaginal pH<br>Culture	Nugent *et al.* (1991) [9]	Two cycles (8 weeks)	Each day	What should be regarded as normal vaginal flora?<br><br>Is the finding of BV, Candida spp., *M. hominis*, *U. urealyticum*, or ‚B-haemolytic streptococci on a single occasion in asymptomatic women significant?<br><br>Is the finding of normal VMF on a single occasion in symptomatic women significant?”
**Keane *et al.* (1997) [2]**	Non pregnant healthy women	Vaginal self swabs	21	Gram stain<br>Vaginal pH	Ison and Hay (2002) [10]	One cycle (25 to 35 days)	Each day	3 groups were found: A: Normal VMF throughout, B: Abnormal VMF throughout, C: Normal VMF in the beginning and then a transient shift to intermediate VMF or BV.<br><br>The existence of group C was surprising. The onset of abnormal VMF in this group occurred mainly in the first part of the MC.<br><br>pH values did not always rise throughout the menses and elevated values were also found in groups A and C which did not correspond to the presence or subsequent development of abnormal VMF.<br><br>In group A and C there was a trend for the numbers of Lactobacilli to fluctuate throughout the MC.<br><br>The use of OC did not appear to influence the Lactobacilli concentration in group A.<br><br>GPC tended to appear mostly at the beginning of the menstrual cycle in groups A and C.

Authors	Inclusion criteria	Sample	Subjects	Techniques	Grading	Period	Sampling frequency	Principal findings
**Hay *et al.* (1997) [3]**	Women treated, minimal 3 times, for BV in the previous 18 months and with stable relationships	Vaginal self swabs	18	Gram stain	Nugent *et al.* (1991) [9]	10 days – 10 months	Not specified	Whether antifungal treatment was taken or not, in many cases candida arose, to be followed by BV.<br><br>BV developed within 48 h of unprotected intercourse on only one occasion, and resolved after intercourse on 9 occasions.<br><br>In most of the women BV resolved after 3 to 4 days' treatment. Usually an intermediate VMF was present transiently. Relapse could occur within a few days.<br><br>BV arose just before menstruation, or during menstruation more often than mid-cycle; a time when it was more likely to resolve.
**Schwebke *et al.* (1999) [4]**	Premenopausal, nonpregnant women aged >18 years without clinical evidence of genital tract infection, including BV	Vaginal self swabs	51	Gram stain	Nugent *et al.* (1991) [9]	6 weeks	Each day	Maintenance of consistently normal VMF occurred in only a minority of women.<br><br>Transient fluctuations in VMF were common and often marked, however, the incidence of symptomatic BV in this cohort approached 10%.<br><br>Behaviors that were associated with unstable VMF are very similar to those that are associated with BV and STDs.<br><br>There was no correlation between use of hormonal contraception and changing flora.<br><br>An association between receptive oral sex and unstable VF was found. Strong similarities exist between the anaerobic bacteria associated with gingivitis and those associated with BV.<br><br>Use of vaginal medication and the occurrence of menses were the only 2 diary variables associated with adverse changes in VMF the day after the behavior.<br><br>Women who reported in their enrollment interview a greater number of sex partners in the past 12 months, more frequent episodes of vaginal intercourse, less frequent use of condoms, and use of spermicide were more likely to exhibit day-to-day changes in their VMF

Authors	Inclusion criteria	Sample	Subjects	Techniques	Grading	Period	Sampling frequency	Principal findings
**Eschenbach *et al.* (2000) [5]**	18–40 years, regular MCs, no or only 1 sexual partner, and no use of contraception other than condoms	Cervical swabs<br>Vaginal swabs<br>Urine specimen for culture	74	Gram stain<br>Vaginal pH<br>Culture	Nugent *et al.* (1991) [9]	1 cycle (25 to 35 days)	3 moments each:<br>menstrual phase (days 1–5)<br>preovulatory phase (days 7–12)<br>postovulatory phase(days 19–24)	The number of subjects with high concentrations of *Lactobacillus* increased significantly over the menstrual cycle independent of the presence of BV, in contrast, the concentration of non-*Lactobacillus* species tended to be higher at menses.<br><br>A statistically significant linear decrease occurred in the recovery of *Prevotella* species from subjects without BV over the menstrual cycle.<br><br>Most VMF microorganisms were maintained at a rather stable rate over these 3 points of the menstrual cycle.<br><br>*C. albicans* was associated with both vulvar erythema and pruritus on days 19–24 of the menstrual cycle.<br><br>The volume of vaginal discharge increased and the amount of cervical mucus decreased over the menstrual cycle.
**Morison *et al.* (2005) [6]**	Asymptomatic, married, using no contraception, regular MC	Vaginal self swabs	30	Gram stain	Nugent *et al.* (1991) [9]	Four cycles	Alternate days	Similar transient fluctuations over the menstrual cycle were found to those in industrialised countries.<br><br>There was no evidence that sexual intercourse was associated with increased frequency of BV.<br><br>The data did not support the hypotheses that menstrual hygiene materials might explain the high prevalences of BV found in sub-Saharan Africa compared to industrialised countries.<br><br>We found a high degree of fluctuation in women's patterns of vaginal flora over time.<br><br>We also did not find any association between frequency of intercourse and BV in the following menstrual cycle.

Authors	Inclusion criteria	Sample	Subjects	Techniques	Grading	Period	Sampling frequency	Principal findings
**Brotman *et al.* (2010) [7]**	Non-pregnant women who reported the use of vaginal douche products in the 2 months before screening	Vaginal self swabs	39	Gram stain	Nugent *et al.* (1991) [9]	Four cycles (16 weeks)	Twice weekly	Rapid fluctuations in vaginal bacterial communities were common.<br><br>Women may have short episodes of bacterial vaginosis that spontaneously resolve without antibiotic treatment.<br><br>Longitudinal studies may miss bacterial vaginosis episodes if samples are collected infrequently.<br><br>Recent report of lubricant use and rectal sex were associated with incident bacterial vaginosis as defined by Gram stain.
**Srinivasan *et al.* (2010) [8]**	No specific inclusion criteria are given	Vaginal self swabs	22	Gram stain, qPCR (11 species) women with BV + 5d metronidazole	Nugent *et al.* (1991) [9]	7-14d<br>+ at 2 weeks<br>3 and 4 weeks	Each day (7-14) + at 3 additional moments	The microbiota of the human vagina can be highly dynamic.<br>Healthy women are colonized with Lactobacillus species, but levels can change dramatically over a month.<br><br>An increase in *G. vaginalis* levels associated with menses, accompanied by decreased quantities of *L. jensenii* and *L. crispatus* were observed<br><br>Participants with BV have diverse communities of fastidious bacteria that are depleted by vaginal metronidazole therapy.<br>Women with recurrent BV initially respond to antibiotic treatment with steep declines in bacterial concentrations, but these bacteria later reemerge, suggesting that antibiotic resistance in these bacteria is not an important factor mediating BV recurrence.<br><br>Recurrence of BV is associated with reappearance of BV-associated bacteria suggesting re-infection or resurgence from an endogenous reservoir.<br><br>The rate of decrease of the BV bacteria with antibiotic treatment varies, suggesting that longer antibiotic treatments may be warranted in some women.

Authors	Inclusion criteria	Sample	Subjects	Techniques	Grading	Period	Sampling frequency	Principal findings
**Present study**	Non pregnant women with a regular MC and no use of contraception, except condoms.	Vaginal self swabs	17	Gram stain<br>Culture<br>Vaginal pH	Verhelst *et al.*, (2005) [11]	Two cycles (8 weeks)	Each day<br>Each week<br>At first and last visit<br>Each day	There is a huge interindividual variability of predominant VMF present, however, the VMF of most subjects was rather stable.<br><br>Besides Lactobacilli, GPC are important members of the VMF.<br><br>*L. crispatus* was the species that was most negatively affected by the menses, the presence of the other lactobacilli did not change much.<br><br>The increased presence of GPC seems to be the most characteristic feature in common to all disturbances of the VMF.<br><br>There is a possible existence of an equilibrium between lactobacilli and GPC in normal VMF, which can be disturbed by menses and SI.<br><br>Grade Ib may be an intermediate VMF, because it often followed or preceded grade II or IV VMF.<br><br>Occasional sampling may miss episodes of BV or candidiasis and on the other hand may overemphasize the importance of an occasionally single positive swab.

References

1. Priestley CJ, Jones BM, Dhar J, Goodwin L (1997) What is normal vaginal flora? Genitourin Med 73(1):23-28.

2. Keane FE, Ison CA, Taylor-Robinson D (1997) A longitudinal study of the vaginal flora over a menstrual cycle. Int J STD AIDS 8(8):489-494.

3. Hay PE, Ugwumadu A, Chowns J (1997) Sex, thrush and bacterial vaginosis. Int J STD AIDS 8(10):603-608.

4. Schwebke JR, Richey CM, Weiss HL (1999) Correlation of behaviors with microbiological changes in vaginal flora. J Infect Dis 180(5):1632-1636.

5. Eschenbach DA, Thwin SS, Patton DL, Hooton TM, Stapleton AE, et al. (2000) Influence of the normal menstrual cycle on vaginal tissue, discharge, and microflora. Clin Infect Dis 30(6):901-907.

6. Morison L, Ekpo G, West B, Demba E, Mayaud P, et al. (2005) Bacterial vaginosis in relation to menstrual cycle, menstrual protection method, and sexual intercourse in rural Gambian women. Sex Transm Infect 81(3):242-247.

7. Brotman RM, Ravel J, Cone RA, Zenilman JM (2010) Rapid fluctuation of the vaginal microbiota measured by Gram stain analysis. Sex Transm Infect 86(4):297-302.

8. Srinivasan S, Liu C, Mitchell CM, Fiedler TL, Thomas KK, et al. (2010) Temporal variability of human vaginal bacteria and relationship with bacterial vaginosis. PLoS One 5:e10197.

9. Nugent RP, Krohn MA, Hillier SL (1991) Reliability of diagnosing bacterial vaginosis is improved by a standardized method of gram stain interpretation. J Clin Microbiol 29(2):297-301.

10. Ison CA, Hay PE (2002) Validation of a simplified grading of Gram stained vaginal smears for use in genitourinary medicine clinics. Sex Transm Infect 78(6):413-415.

11. Verhelst R, Verstraelen H, Claeys G, Verschraegen G, Van Simaey L, et al. (2005) Comparison between Gram stain and culture for the characterization of vaginal microflora: definition of a distinct grade that resembles grade I microflora and revised categorization of grade I microflora. BMC Microbiol 5:61.
