# Supplementary material for: Longitudinal Study of the Dynamics of Vaginal Microflora during Two Consecutive Menstrual Cycles
Source: PLoS One. 2011 Nov 30;6(11):e28180. doi: 10.1371/journal.pone.0028180 (PMC3227645; doi:10.1371/journal.pone.0028180)
Supplement: File S2 — Bacterial species cultured from at least 10% of all samples of at least one of the VMF grades. (PDF) [file pone.0028180.s002.pdf]

Supporting Information file 2.

VMF Grade	Ia	Iab	Ib	I-like	0	II	III	IV	Menses	Total
**Number of culturing moments**	**59**	**10**	**30**	**9**	**2**	**30**	**27**	**11**	**29**	**178**
**Number of women from the N and D group (N/D)**	**8/2**	**4/0**	**3/4**	**2/2**	**0/2**	**4/5**	**1/6**	**2/2**	**8/7**	**9/8**
**Species**										
**Lactobacillus spp.**										
Lactobacillus coleohominis	16.9					3.3	3.7			6.7
Lactobacillus crispatus	76.3	90.0	13.3	11.1		46.7	3.7	9.1	24.1	42.1
Lactobacillus gasseri	8.5	40.0	43.3			23.3		9.1	13.8	16.9
Lactobacillus iners	11.9	20.0	56.7	11.1		23.3	29.6	9.1	17.2	24.2
Lactobacillus jensenii	52.5	70.0	23.3	22.2		50.0	14.8		34.5	37.1
Lactobacillus vaginalis	13.6	30.0	23.3	11.1		23.3	3.7			15.2
**Bifidobacterium spp.**										
Bifidobacterium breve	3.4			11.1						1.7
Bifidobacterium dentium				22.2						1.1
**BV-related species (excluding those mentioned in GPC)**										
Aerococcus christensenii						3.3	18.5		6.9	3.4
Gardnerella vaginalis	10.2	10.0	16.7	11.1		60.0	55.6	9.1	27.6	26.4
Prevotella amnii							14.8		6.9	2.2
Prevotella bivia				22.2		6.7	18.5		3.4	5.1
Prevotella disiens	1.7		16.7			3.3			3.4	3.9
Prevotella melaninogenica				11.1						0.6
Prevotella timonensis			10.0			6.7			6.9	2.8
**Gram Positive cocci**										
Anaerococcus murdochii		10.0								0.6
Anaerococcus tetradius	1.7						33.3	9.1	6.9	6.2
Anaerococcus vaginalis	3.4		6.7	11.1		13.3	3.7		10.3	5.6
Enterococcus faecalis	8.5	10.0	6.7	55.6	50.0	3.3	3.7	63.6	17.2	12.9
Finegoldia magna	6.8	10.0	23.3	33.3	100.0	13.3	14.8	27.3	27.6	15.7
Peptoniphilus harei		20.0								1.1
Peptoniphilus lacrimalis	1.7	10.0	10.0			3.3	3.7		6.9	3.9
Peptoniphilus sp.		10.0						9.1	3.4	1.1
Peptostreptococcus anaerobius	8.5	20.0	23.3	11.1		36.7	44.4	18.2	41.4	22.5
Peptostreptococcus asaccharolyticus	30.5	60.0	56.7	33.3		60.0	37.0	45.5	41.4	43.3
Staphylococcus aureus			6.7	11.1		10.0	11.1	9.1	10.3	5.6
Staphylococcus epidermidis	22.0	40.0	23.3		50.0	16.7	22.2		24.1	20.2
Staphylococcus haemolyticus	1.7	10.0	6.7				3.7	9.1	3.4	3.4
Streptococcus agalactiae	3.4	40.0	3.3	11.1		20.0		18.2	20.7	9.0
Streptococcus anginosus Group	32.2	30.0	30.0	33.3		46.7	59.3	72.7	55.2	40.4
Streptococcus cristatus				11.1						0.6
Streptococcus mitis				11.1			3.7		3.4	1.1

**VMF Grade**	**Ia**	**Iab**	**Ib**	**I-like**	**0**	**II**	**III**	**IV**	**Menses**	**Total**
**Number of culturing moments**	**59**	**10**	**30**	**9**	**2**	**30**	**27**	**11**	**29**	**178**
**Number of women from the N and D group (N/D)**	**8/2**	**4/0**	**3/4**	**2/2**	**0/2**	**4/5**	**1/6**	**2/2**	**8/7**	**9/8**
**Species**										
Streptococcus sp.				11.1						0.6
**Other Species**										
Actinomyces urogenitalis			6.7				7.4	9.1	10.3	2.8
Alloscardovia omnicolens	5.1	20.0	33.3			6.7	3.7	81.8	17.2	15.2
Atopobium parvulum			3.3		50.0		3.7		3.4	1.7
Clostridium saccharobutylicum		10.0								0.6
Corynebacterium coyleae		10.0				3.3				1.1
Corynebacterium glucuronolyticum		10.0								0.6
Escherichia coli	5.1		23.3	33.3	50.0	6.7	3.7	36.4	17.2	11.8
Fusobacterium nucleatum		10.0				6.7	7.4			2.8
Klebsiella pneumoniae	1.7		3.3	11.1			3.7	18.2		3.4
Propionibacterium avidum	6.8	20.0		11.1		3.3				4.5
Veillonella montpellierensis		10.0				6.7			3.4	1.7
Veillonella parvula	3.4		6.7	22.2		3.3		18.2	6.9	5.1
